# Identification of *Peptoniphilus harei* From Blood Cultures in an Infected Aortic Aneurysm Patient: Case Report and Review Published Literature

**DOI:** 10.3389/fcimb.2021.755225

**Published:** 2021-12-22

**Authors:** Xue Wan, Shuang Wang, Min Wang, Jinhua Liu, Yu Zhang

**Affiliations:** ^1^Laboratory Department of Clinical Laboratory, The Second Hospital of Jilin University, Changchun, China; ^2^Department of Dermatology, The Second Hospital of Jilin University, Changchun, China; ^3^Department of General Surgery, The Second Hospital of Jilin University, Changchun, China; ^4^Changchun Customs Technology Center, Changchun, China

**Keywords:** bacteremia, gram-positive anaerobic cocci, *Peptoniphilus harei*, MALDI–TOF, 16s rDNA sequencing

## Abstract

Gram-positive anaerobic cocci (GPAC) are a commensal part of human flora but are also opportunistic pathogens. This is possibly the first study to report a case of *Peptoniphilus harei* bacteremia in an abdominal aortic aneurysm (AAA) patient. Matrix-assisted laser desorption ionization time-of-flight mass spectrometry (MALDI-TOF MS) failed to identify the isolate and molecular analysis confirmed it as *P. harei*. A comprehensive literature review revealed that *P. harei* is an emergent pathogen. This study serves as a reminder for practicing clinicians to include anaerobic blood cultures as part of their blood culture procedures; this is particularly important situations with a high level of suspicion of infection factors in some noninfectious diseases, as mentioned in this publication. Clinical microbiologists should be aware that the pathogenic potential of GPAC can be greatly underestimated leading to incorrect diagnosis on using only one method for pathogen identification. Upgradation and correction of the MALDI-TOF MS databases is recommended to provide reliable and rapid identification of GPAC at species level in medical diagnostic microbiology laboratories.

## Introduction

Gram-positive anaerobic cocci (GPAC), a large group of anaerobic bacteria, comprise several bacterial genera. Although they are members of the normal microbiota, they are also known to be important pathogens causing human diseases, accounting for approximately 25%-30% of all isolated anaerobic bacteria from clinical specimens (Murphy and Frick, 2013). Despite the advances in medical practice in recent years, it is well known that the presence of anaerobes in the bloodstream continues to be associated with high mortality and necessitates appropriate treatment (Cobo et al., 2020; Gajdács and Urbán, 2020). The early identification of anaerobes is important for the initiation of appropriate treatment in patients with bacteremia, and the correct identification up to the species level of GPAC often helps to distinguish between clinically relevant microorganisms and culture contaminants. However, the culture and identification of many GPAC strains in diagnostic laboratories remain hampered due to the requirement of prolonged incubation times and time-consuming phenotypic identification procedures (Simmon et al., 2008; Murphy and Frick, 2013; De Keukeleire et al., 2016; Badri et al., 2019). Following the introduction of 16S rRNA for determining the phylogenetic relationships among prokaryotes, this method has been regarded as the “gold standard” for both microbial phylogeny and ecological studies (Prakash et al., 2013) and is also considered a valuable tool for assigning species to newly isolated bacterial strains. Recently, a new method has been positioned at the forefront for bacterial identification in the future–matrix-assisted laser desorption ionization time-of-flight mass spectrometry (MALDI-TOF MS) (La Scola et al., 2011; Veloo et al., 2011a). At present, two MALDI-TOF MS systems are commercially available for routine use: Bruker Biotyper and VITEK MS. The methodology of the two systems is similar, but they contain differences in the composition of databases and the application of software packages for data analysis, as well as Bruker Biotyper provides results in a log(score), while VITEK MS provides results in percentage of similarity (Lee et al., 2017).

*Peptoniphilus* genus are GPAC that were formerly classified in the genus *Peptostreptococcus*, currently consisting of 17 valid published species (http://www.bacterio.net/peptoniphilus.html) (Ezaki et al., 2001), which are involved in various opportunistic human infections mainly as part of polymicrobial infections. The diagnosis of *Peptoniphilus* infection is mainly based on culture, and the identification of strains is usually performed using phenotypic tests, MALDI-TOF MS, and/or molecular methods (Murphy and Frick, 2013). However, many laboratories do not yet have the capacities to use more current (i.e. MALDI-TOF MS and 16S rRNA sequence) technologies in their diagnostic workflow, therefore they heavily rely on biochemical tests, thus *P. harei* is often misidentified as *P. asaccharolyticus*, as these two species have the same biochemical characteristics and cannot be differentiated from each other phenotypically (Veloo et al., 2011b). In this study, we report a patient who presented with AAA and was found to have anaerobic bacteremia that was initially missed because anaerobic blood cultures had been eliminated from the standard blood culture protocol of the institution where he was first assessed. Unfortunately, when he was hospitalized again, Bruker Biotyper and VITEK MS failed to identify GPAC at the species level. The isolate was successfully identified using 16S rRNA sequence analysis and a phylogenetic tree was drawn. We supplemented the identification of this isolate with a literature search into previous clinical cases of *P*. *harei* and *P. asaccharolyticus* infections.

## Materials and Methods

### Case Report

**Figure 1:**
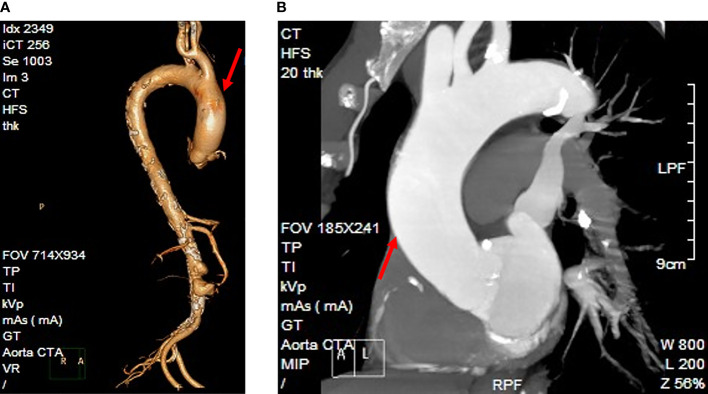
The patient’s diagnostic imaging. **(A)** Reconstruction of the CT angiography image. **(B)** CT angiography indicated an unruptured abdominal aortic aneurysm (arrowhead).

**Figure 2:**
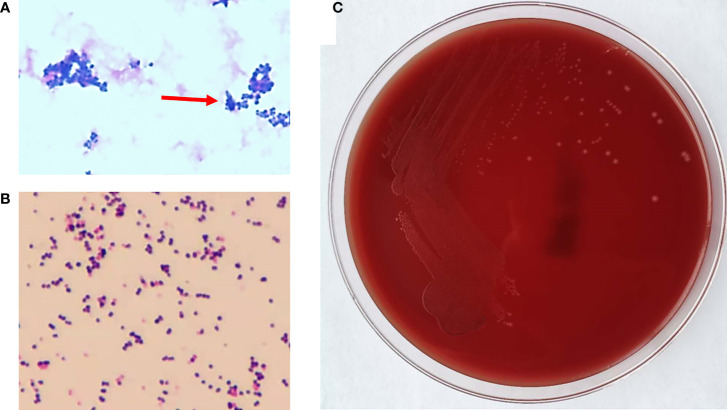
The phenotype of *Peptoniphilus harei*. **(A)** Gram staining of *P. harei* from a positive aerobic blood culture showed gram-positive cocci (1,000× magnification) (arrowhead). **(B)** Gram-stained colonies showing gram-positive cocci. **(C)** *P. harei* colonies on blood agar after 48 h of incubation under anaerobic conditions.

A 75-year-old man presented to the emergency department (ED) of the Second Hospital of Jilin University (Changchun, China) with pain in his right lower extremity. His past medical history was notable for gastric cancer 10 years prior to the present admission. Four months before the current presentation, the patient was admitted to our institution because of hematemesis and melena, although CT angiography (CTA) showed the presence of an unruptured AAA (**Figure 1**), which was treated with endovascular stent–graft implantation. Subsequently, the patient improved clinically, but exhibited unexplained persistent bloody stools and was discharged on day 12 of admission. On the present admission, the patient presented with right lower extremity edema and a bruised with no obvious cause. The patient had no diabetes or coronary heart disease. CTA showed severe stenosis or occlusion of the right lower-extremity artery and his blood chemistries were remarkable; the initial laboratory studies were significant for elevated white blood cells at 17.6 × 10^9^/L (reference range, 3.5−9.5 × 10^9^/L), 93.4% neutrophils (reference range, 40%−75%), a serum C-reactive protein (CRP) level of 194 mg/L (reference range, 0.2−7.44 mg/L), and procalcitonin at 9.39 ng/ml (reference range, <0.05 ng/ml). He was empirically treated with cefminox (1000 mg/i.v/12 h) and admitted for further management of suspected lower-limb ischemia. On day 2 of admission, the patient complained of severe pain in the right lower extremity. On physical examination, the skin defect detected on the proximal part of the great right toe was about 2.0 × 2.5 cm in size with liquefied necrotic tissue. A wound swab was collected from a chronic ulcer on his right foot and 40 mL of blood was collected using a syringe. The blood was cultured using a BacT/Alert three-dimensional (bioMérieux Inc.; Durham, NC) automated blood culture system. Each blood culture consisted of a set of two (FA Plus aerobic, FN Plus anaerobic) bottles. Two sets of blood samples were collected from the patient. The only positive microbiological test was *Streptococcus anginosus* from the wound secretion, and drug susceptibility testing by disk diffusion (Oxoid, Hampshire, United Kingdom) according to the Clinical and Laboratory Standards Institute (CLSI) recommendations revealed that the isolate was resistant to erythromycin, clindamycin, and tetracycline, and susceptible to cefepime, levofloxacin, linezolid, and vancomycin. These two anaerobic blood cultures were positive after 3 days of incubation, and the broths were subcultured onto Columbia agar plates (Oxoid, Thermo Scientific, Hampshire, United Kingdom) that were incubated anaerobically. The aerobic medium bottles remained negative. Within 1 day, growth on Columbia agar plates revealed pinpoint, smooth glistening gray colonies and Gram staining showed gram-positive cocci (**Figure 2**). Subsequently, the patient’s antimicrobial therapy was switched to moxifloxacin (400 mg/i.v/24 h) and penicillin G (320 million U/iv/6 h). The bacteremia was ultimately cleared after an additional 5 days, and the patient was discharged home on penicillin G for 2 weeks. Surveillance blood cultures performed 2 weeks after the completion of therapy were negative and the patient had improved clinically.

### Identification by MALDI- TOF

Pure colonies of the causative bacteria for bacteremia were selected for identification by two MALDI–TOF MS systems, Microflex LT instrument and MALDI Biotyper 3.1 software (Bruker Daltonics, Bremen, Germany) and Vitek MS RUO IVD library (v3.2) (bioMérieux, Marcy I’Étoile, France) in accordance with the manufacturers’ recommendations. Bruker Biotyper reports the identification of each organism with a numerical score, and scores of ≥2.0 and ≥1.7 represent acceptable probable species-and genus-level identifications, respectively. VITEK MS analysis produces a confidence value, reported as a percentage up to 99.9%. The return of an identification of a single taxon, regardless of the confidence value, is considered an acceptable level of identification. If no identification is provided, the isolate is considered unidentified.

### Molecular Investigations

16S rRNA sequencing is a valuable tool for definitive molecular identification of important clinical isolates that cannot be readily identified by phenotypic methods or MALDI-TOF. The identity of the clinical isolate was further confirmed by amplifying the 16S rRNA gene with the universal primers 27F and 1492R (Lane, 1991). Next, independent phylogenetic analyses of the partial 16S rDNA sequences were performed using the Neighbor-Joining method (Saitou and Nei, 1987) with the MEGA 6 software (Molecular Evolutionary Genetics Analysis Version 6.0) (Tamura et al., 2013), and the robustness of the trees was evaluated by 1,000 bootstrap replicates to infer the evolutionary history.

### Antimicrobial Susceptibility Testing

The minimum inhibitory concentrations (MICs) of various antibiotics against the isolated organisms were determined using the E-test method and according the 2021 CLSI criteria.

### Literature Search

After the species identity of the causal organism was confirmed, an electronic search was performed using the key words “*Peptoniphilus harei*”, “*Peptoniphilus asaccharolyticus*”, and “human infection” in the PubMed database.

## Results

**Figure 3:**
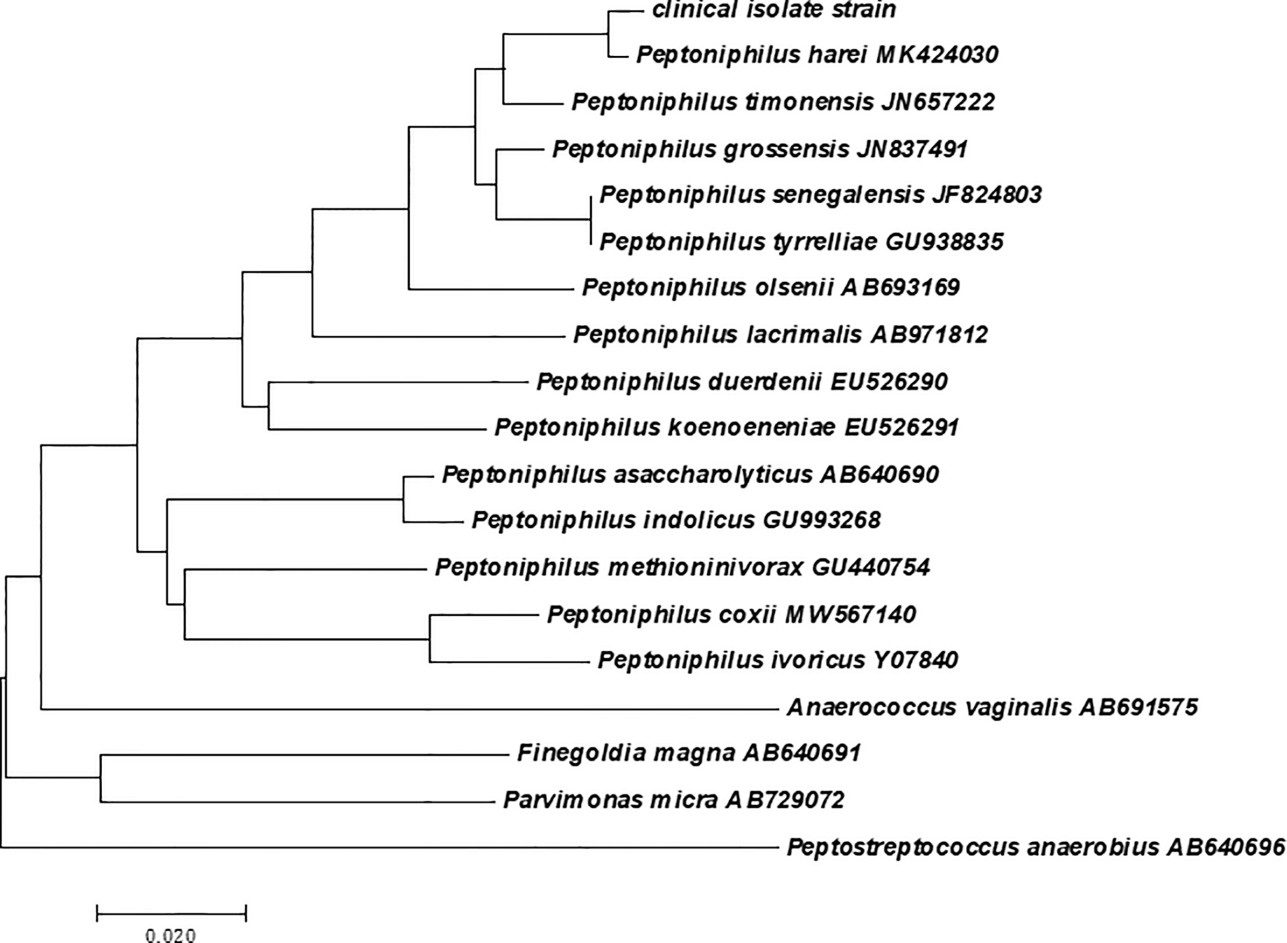
Phylogenetic analysis based on the 16S rRNA gene sequence involving the clinical isolate strain, and the phylogram was constructed using the neighbor-joining method with 1000 bootstrap replicates.

Initially, small colonies were observed in pure culture and were subsequently analyzed *via* the Bruker Biotyper system. The provided identification of *P. harei* yielded a log score of 1.288, according to Bruker recommendations; however, no reliable identification at genus level (log score ≥1.70) or species level (log score ≥2) was observed with the most recent MALDI Biotyper database (i.e., the MALDI Biotyper database was not able to correctly identify the isolate). The results of mass spectrometry are dependent on the quantity and accuracy of the database; therefore, we reidentified the isolate by Vitek MS as *P. asaccharolyticus* based on a confidence level of 99.9%. To our surprise, when additional molecular identification *via* PCR amplification of the 16s rRNA gene, which facilitates the precise identification of the anaerobic bacteria involved, was performed, the clinical isolate exhibited a 16S rRNA similarity of 99% with *P. harei* (GenBank NR_026358.1). The sequence has been deposited in GenBank under accession number MZ008068. A multiple alignment was created from the consensus sequences of the novel strains as well as the type sequences of other GPACs with nomenclature obtained from NCBI GenBank. A phylogenetic tree (**Figure 3**) of 19 nucleotide sequences was constructed. Consistent with the sequencing results, the clinical isolate strain clustered most closely with *Peptoniphilus harei* MK404230. Therefore, the clinical isolate strain was confidently identified as *P. harei*. The strain was susceptible to all antimicrobials tested and the MICs obtained for this strain were as follows: penicillin G (0.032 μg/mL), ampicillin (0.032 μg/mL), clindamycin (0.064 μg/mL), meropenem (0.008 μg/mL), ceftriaxone (0.125 μg/mL), chloramphenicol (1.5μg/mL) and metronidazole (0.064μg/mL).

In a comprehensive literature search, we found that the isolation of *P. harei* in human samples has been mainly observed in the context of polymicrobial infections and that *P. harei* is an uncommon cause of infection as a single agent. Walter et al. (Walter et al., 2014) identified six strains of *P. harei* and six strains of *P. asaccharolyticus* from anaerobic bone and joint infection samples using Biotyper 3.0 software. Cobo et al. employed a Bruker system to identify a case of peritoneal infection (Cobo et al., 2017) and lymphocele infection (Cobo, 2018) due to *P. harei*, with scores of 2.27 and 2.09, respectively. Another study described a case of *Escherichia coli* and *Peptoniphilus* spp. mixed osteomyelitis occurring after trauma; however, the score given by the Bruker system in this case was 1.71, and no additional identification method, such as PCR amplification of the 16S rRNA gene, was used (Costescu Strachinaru et al., 2020). Three strains had been isolated from abscesses in different locations as part of mixed polymicrobial infections (Abdulrahman and Gateley, 2015; Parha et al., 2015; Le Bihan et al., 2019). *P. harei* is considered a rare pathogen of severe infections, such as infections of the periprosthetic joint (Rieber et al., 2020), breast implants (Seng et al., 2015), or endometrioma (Cornman-Homonoff et al., 2019). Interestingly, in most *P. harei* cases, species identification was performed using Bruker Biotyper, but infections due to *P. asaccharolyticus* were diagnosed using Vitek MS (Muller-Schulte et al., 2019). The essential data from some *P. harei* infections are summarized in **Table 1**.

**Table 1:** Cases of *P. harei* infection in humans reported in the literature.

Report year	Patient age/gender	Country	Diagnosis	Identification by MALDI- TOF	Molecular investigations	Type of infection
2020	57/M	Belgium	Osteomyelitis	Bruker Biotyper (score: 1.71)	NO	Polymicrobial
2020	84/M	Germany	Periprosthetic joint infection	Bruker biotyper	16S rRNA partial gene sequence	Polymicrobial
2019	43/F	UK	Breast abscess	MALDI-TOF MS	NO	Polymicrobial
2019	40/F	USA	Infected endometrioma	Unknown	Unknown	Monomicrobial
2017	60/M	Spain	Lymphocele infection	Bruker Biotyper (score: 2.09)	NO	Monomicrobial
2017	48/F	Spain	Peritoneal infection	Bruker Biotyper (score 2.27)	NO	Monomicrobial
2015	68/F	UK	Brain abscess	MALDI-TOF MS	16S rDNA partial sequencing	Polymicrobial
2015	22/F	UK	Primary actinomycosis of the breast	NO	NO	Polymicrobial

## Discussion

Although anaerobic bacteria are common pathogens, they are an uncommon cause of bacteremia in humans. A significant minority of bacteremia cases are caused by obligate anaerobic organisms, which are associated with poor clinical outcomes and high rates of mortality. In the first and only case series of *Peptoniphilus* bloodstream infections presented in 2014, 3 of the 15 cases had a fatal outcome (Brown et al., 2014). *P. harei* was firstly isolated by Murdoch et al. in 1998 from the pus of various infected sites (Murdoch et al., 1997). It is an uncommon cause of infection as a single agent and is mainly observed in polymicrobial infections in human samples, including pressure ulcers, osteoarticular samples, and skin and soft-tissue infections. Our study was the first report of *P. harei* bacteremia in a patient with AAA.

The correct identification of pathogens is of paramount importance in clinical practice, epidemiological studies, and the basic research of microorganisms. Previous studies have shown that MALDI-TOF-MS is satisfactory for the genus identification of clinically pathogenic anaerobic bacteria; however, this method still suffers from drawbacks in the precise identification of rare anaerobes at species level. A meta-analysis that included 28 studies covering 6,685 strains of anaerobic bacteria showed that the identification accuracy of MALDI-TOF MS was 84% for species and 92% for genera, meanwhile, the identification accuracy rate was 90% for VITEK MS and 86% for MALDI Biotyper system (Li et al., 2019). A multi-center study that assessed the clinical performance of the VITEK MS system in the identification of anaerobic bacteria found that 651 anaerobic bacterial isolates representing 11 genera and 26 separate species (a total of 91.2%; 594/651) were correctly identified to species level as confirmed by 16S rRNA gene sequencing (Garner et al., 2014). However, Ferrand et al. (2018) reported that the Biotyper system identified more isolates belonging to less commonly encountered anaerobic and facultative anaerobic organisms, including *Peptoniphilus*, than the Vitek MS system. In this study we observed that the species level was successfully identified by neither of these two systems, although both included *P. harei* and *P. asaccharolyticus* in their database. As mentioned earlier, invasive anaerobic infections are life-threatening, and the mortality rate of anaerobic bacteremia can be as high as 40%. Meanwhile, it is difficult to identify rare or newly identified species using conventional phenotyping methods and commercial kits. The causes of the lack of identification by Bruker Biotyper TOF-MS and the erroneous identification by Vitek MS are mainly due to the lack of software or updates in data library databases. It should be mentioned that although the European Network for the Rapid Identification of Anaerobes (ENRIA) project in recent years has successfully increased the number of anaerobic isolates that can be identified with high confidence (Veloo et al., 2018). But the accuracy of both systems should be further increased by expanding, updating, and perfecting the databases.This is essential for improving the identification rate and decreasing misidentifications of *Peptoniphilus* isolated from clinical specimens and, subsequently, to improve the reporting of uncommon GPAC infections in humans. Hence, we hypothesized that infection due to *P. harei* may be seriously underestimated depending on the database of the MALDI-TOF MS system, which may allow for additional tests for species identity confirmation.

Our case had several unique clinically interesting characteristics. First, the patient was diagnosed with an AAA 4 months prior to the current presentation. Anaerobic bacteria are an uncommon but important cause of AAA, and various anaerobic bacteria have been found in MAAs (Itzhak and Brook, 2009). MAAs are predominant among males older than 60 years, and especially in those older than 65 years (Wang et al., 1996). The present case of a 75-year-old man who had a history of gastric cancer and surgery exhibited the most significant risk factors. Based on a previous imaging study, the presence of inflammation, and persistent bloody stool of unknown cause, it is possible that the original aneurysm was a mycotic aneurysm that remained undiagnosed because of the lack of an aerobic blood culture. Within the past two decades, the incidence of anaerobic bacteremia among hospitalized patients is thought to have decreased because of the implementation of the recommendation to discontinue routine anaerobic blood cultures in some hospitals. We believe that our case illustrates the need for awareness among infectious disease clinicians and other researchers of the continued importance of anaerobic cultures; we propose that they should be specifically requested in situations of a high level of suspicion of the contribution of anaerobes to the infectious process, and that it is necessary to consider MAAs when diagnosing masses in the context of AAA. In addition, *P. harei* is generally considered a co-occurring obligate anaerobe in chronic wounds or diabetic ulcers. Our findings were astonishing because the open wound located on the patient’s right great toe was thought to be the source of *S. anginosus* infection exclusively. Clinicians should consider the independent hematogenous spread of *P*. *harei* and *P*. *harei*-associated mono-bacteremia in cases of patients with chronic wound infections, as in the present case.

In summary, we report this case to increase the public awareness of *P*. *harei* infections, *P. harei* bacteremia is an uncommon but important human infection, routine use of anaerobic blood cultures can contribute to the diagnosis of a significant number of anaerobic bacteremia cases, resulting in the opportunity to better manage the infections. GPAC have a high pathogenic potential in more severe, invasive infections. To improve reliable identification of GPAC using a combination of multiple technologies will help to better estimate its real prevalence and pathogenicity in a clinical setting.

## Data Availability Statement

The datasets presented in this study can be found in online repositories. The names of the repository/repositories and accession number(s) can be found below: MZ008068.

## Ethics Statement

This work was approved by the Ethics Committee of the Second Hospital of Jilin University. Written informed consent was obtained from the individual(s) for the publication of any potentially identifiable images or data included in this article.

## Author Contributions

XW designed the study, collected the data, and wrote the manuscript. SW and MW were involved in the clinical decision making. JL constructed the tree. YZ reviewed the manuscript and contributed materials. All authors contributed to the article and approved the submitted version.

## Funding

This work was supported by research funds from the Project of the Education Department of Jilin Province, (grant no. JJKH20211145KJ), Jilin Province Science and Technology Project of Traditional Chinese Medicine (grant no. 2020031).

## Conflict of Interest

The authors declare that the research was conducted in the absence of any commercial or financial relationships that could be construed as a potential conflict of interest.

## Publisher’s Note

All claims expressed in this article are solely those of the authors and do not necessarily represent those of their affiliated organizations, or those of the publisher, the editors and the reviewers. Any product that may be evaluated in this article, or claim that may be made by its manufacturer, is not guaranteed or endorsed by the publisher.
